# Data on coincidence site lattice boundaries of abnormally growing Goss grains in Fe-3%Si steel

**DOI:** 10.1016/j.dib.2022.108678

**Published:** 2022-10-15

**Authors:** Tae-young Kim

**Affiliations:** National Center for Standard Reference Data, Korea Research Institute of Standards and Science, Daejeon 34113, Republic of Korea

**Keywords:** Recrystallization, Texture, Electrical steel, Electron backscatter diffraction (EBSD)

## Abstract

Data on the coincidence site lattice (CSL) boundary relationship between Goss and matrix grains in Fe-3%Si grain-oriented electrical steel are reported herein. First, the matrix grains of the Fe-3%Si grain-oriented electrical steel after primary recrystallization were investigated to examine the CSL boundary relationship between major orientations namely, Goss (90°, 45°, 90°), Cube (0°, 0°, 0°), Brass (35°, 45°, 0°), and Copper (90°, 35°, 45°). Then, the abnormal growth morphology of the Goss grains was observed ex-situ using electron backscatter diffraction. During its abnormal growth, the Goss grain grew while consuming the matrix grains. However, matrix grains with a CSL boundary relationship with Goss grains did not disappear but remained. The matrix grains that either remained or disappeared when the Goss grains grew abnormally were investigated, and the fraction of matrix grains that had a CSL boundary relationship with the Goss grains was 27.9% among the 523 remaining grains and 12.8% among the 2813 disappearing grains.

**Specifications Table**

**Table:** 

Subject	Metals and Alloys
Specific subject area	Abnormal grain growth morphology, Goss grains, Fe-3%Si steel
Type of data	Image Graph
How the data were acquired	The inverse pole figure (IPF) map was obtained using electron backscatter diffraction (EBSD, Hikari, EDAX, Inc., NJ, USA). To count the remaining or disappearing grains, each matrix grain was numbered, and the number of matrix grains was counted after heat treatment.
Data format	Raw Analyzed Filtered
Description of data collection	The Goss and matrix grains were observed by EBSD after surface polishing of the sampled Fe-3%Si steel.
Data source location	• Institution: Seoul National University • City/Town/Region: Seoul • Country: Republic of Korea
Data accessibility	Repository name: Mendeley Data Title of the dataset: Data on coincidence site lattice boundaries of abnormally growing Goss grains in Fe-3%Si steel Direct URL to data: https://data.mendeley.com/datasets/xhz59bg94f/3 Dataset DOI: 10.17632/xhz59bg94f.3
Related research article	T.-Y. Kim, H.-K. Kim, Y.-K. Jeong, Y.-K. Ahn, H.-S. Shim, D. Kwon, N.-M. Hwang, Ex-situ time sequential observation on island and peninsular grains in abnormally growing Goss grains in Fe–3%Si steel, Met. Mater. Int. 26 (2020) 1200–1206. https://doi.org/10.1007/s12540-020-00728-3[1].

## Value of the Data

• The data on the coincidence site lattice (CSL) boundaries of abnormally growing Goss grains in Fe-3%Si steel can be used to understand and verify the role of CSL boundaries in the mechanism of abnormal grain growth.
• These data can be beneficial to researchers studying abnormal grain growth mechanisms to effectively induce or limit abnormal grain growth.
• The data on CSL boundaries can be reused in studies that promote or restrict abnormal grain growth.

## 1 Data Description

**Fig. 1:**
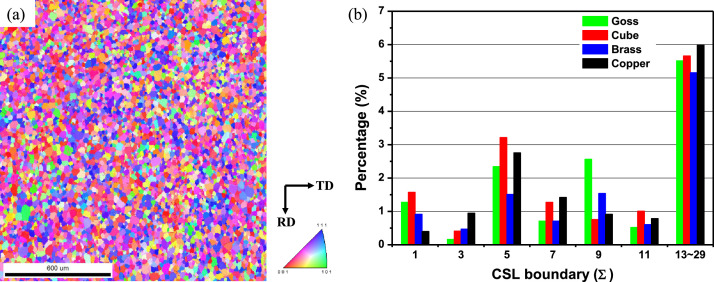
(a) The EBSD IPF maps after primary recrystallization and (b) distribution of CSL boundaries between four types of orientation and matrix grains of primary recrystallization. Four representative orientations are Goss (90°, 45°, 90°), Cube (0°, 0°, 0°), Brass (35°, 45°, 0°), and Copper (90°, 35°, 45°).

Fig. 1a shows an IPF map of the Fe-3%Si grain-oriented electrical steel, for which primary recrystallization was analyzed via EBSD. According to previous studies, the texture of grain-oriented electrical steel after primary recrystallization is mainly composed of {1 1 0} <0 0 1> and {1 1 1} <1 1 2> [2]. This result is confirmed in Fig. 1a. Fig. 1b shows a graph of the distribution of CSL boundaries between the grains in Fig. 1a and four types of orientations. The four typical orientations are Goss (90°, 45°, 90°), Cube (0°, 0°, 0°), Brass (35°, 45°, 0°), and Copper (90°, 35°, 45°). The raw data used for Fig. 1b are deposited in Mendeley Data.

**Fig. 2:**
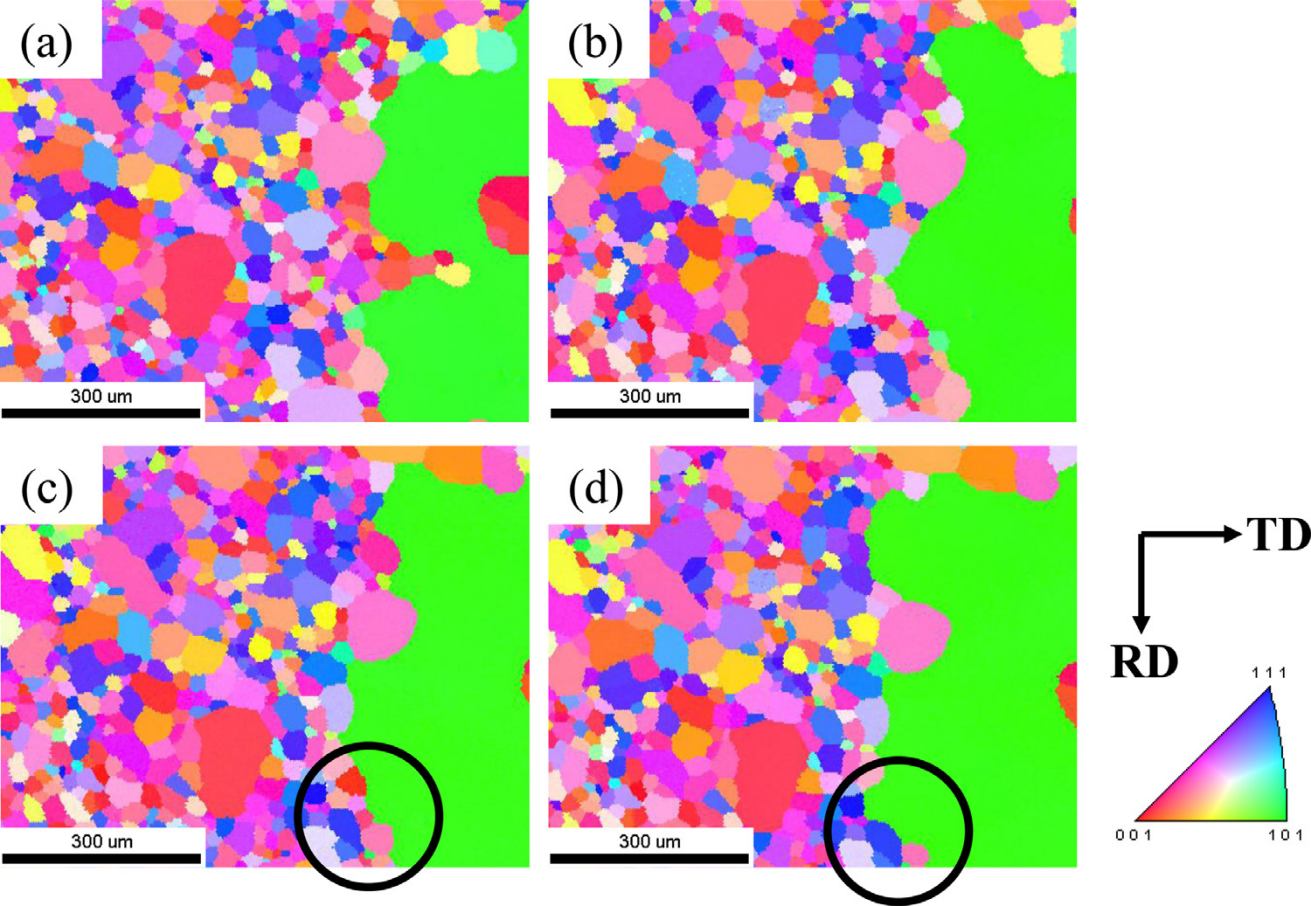
The EBSD IPF maps with time evolution of Goss grains during AGG. (a) The initial state IPF map of the specimen after secondary recrystallization. The IPF maps from (b) to (d) were obtained after annealing at 1080°C sequentially for accumulated times of 11, 13.5, and 16 min, respectively.

**Fig. 3:**
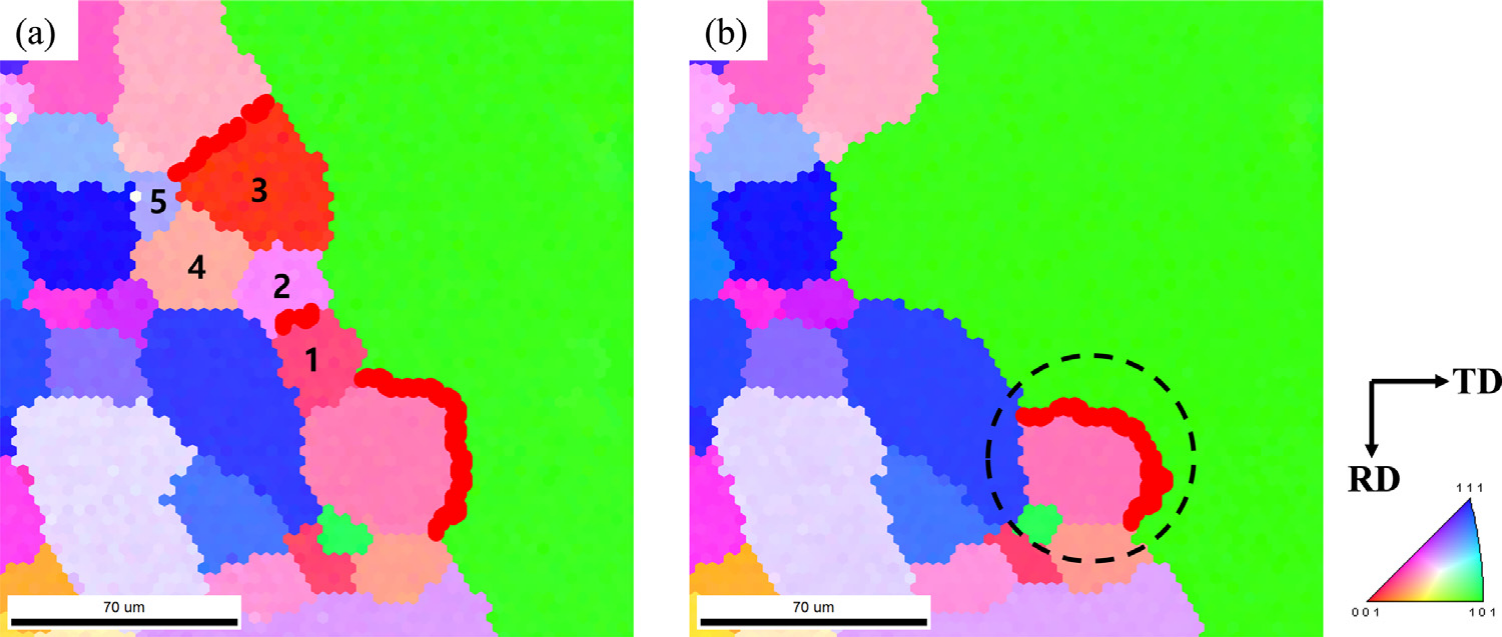
The high magnification microstructures of the area marked by black circles in Fig. 2c and d with accumulated annealing time at (a) 13.5 min and (b) 16min, respectively.

Fig. 2 shows an EBSD IPF map of the abnormal growth of Goss grains in Fe-3%Si grain-oriented electrical steel over time. Fig. 2a shows an IPF map of the initial state of the specimen after secondary recrystallization. The IPF maps shown in Figs. 2b, 2c, and 2d were obtained after sequential annealing for cumulative times of 11, 13.5, and 16 min, respectively. Figs. 3a and 3b show the EBSD IPF maps corresponding to the area marked by black circles in Figs. 2c and 2d, with accumulated annealing times of 13.5 and 16 min, respectively. Fig. 3a shows that the Goss grain meets matrix grains 1, 2, and 3, and the pink grains have a CSL boundary relationship. As shown in Fig. 3b, after the additional heat treatment, matrix grains 1, 2, 3, 4, and 5 are all consumed by the Goss grain, while the pink grains that have a CSL boundary relationship with the Goss grain remain unconsumed.

**Fig. 4:**
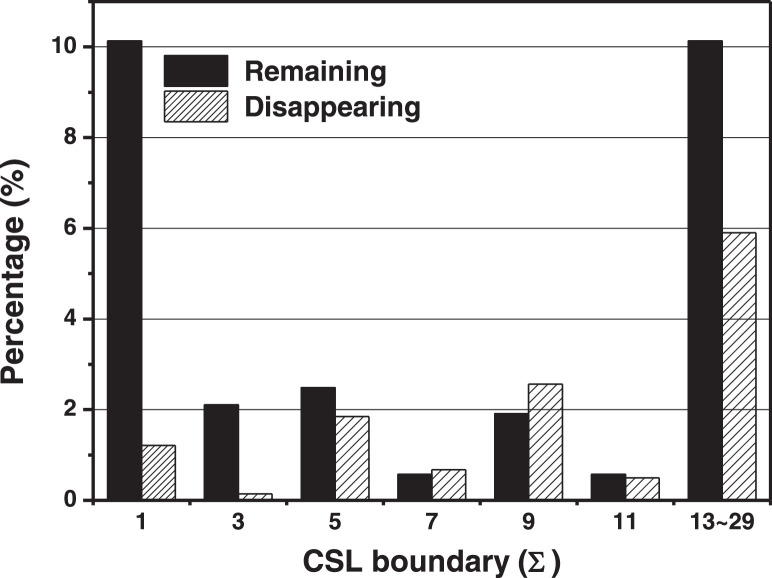
Comparison of CSL boundary distributions between the remaining (black bar) and the disappearing (diagonal pattern bar) grains at the growth front of Goss grains during AGG.

Fig. 4 shows a graph comparing the CSL boundary fraction between the Goss grains and grains that remained or disappeared at the growth front during abnormal grain growth. The remaining or disappearing grains were numbered and counted for comparing the fractions of the CSL boundary in the total of four specimens. In addition to those shown in Fig. 2, the remaining or disappearing grains of three specimens were investigated. Remaining or disappearing grains list 1 refers to the remaining or disappearing grains in Fig. 2, while the remaining or disappearing grains in the other three specimens are summarized in lists 2–4, respectively. The list of remaining or disappearing grains for the four specimens is stored as remaining or disappearing grains lists 1–4 in Mendeley Data. As shown in Fig. 4, the fractions of the CSL boundary with Goss grains were 27.9% and 12.8% among the 523 remaining and 2813 disappearing grains, respectively. The raw data for Fig. 4 are deposited in Mendeley Data.

## 2 Experimental Design, Materials and Methods

Fe-3%Si steel, which is a grain-oriented electrical steel with aluminum nitride added as an inhibitor, was used in the experiment. An ingot of Fe-3%Si steel was hot-rolled to 2.3 mm and cold-rolled to 0.3 mm. Then, the steel was recrystallized at 850°C for 150 s for decarburization and nitriding. To check whether the matrix grains had a relatively high percentage of CSL boundaries for the major orientations—, namely, Goss (90°, 45°, 90°), Cube (0°, 0°, 0°), Brass (35°, 45°, 0°), and Copper (90°, 35°, 45°),—the specimen was polished for microstructure observation and analyzed by EBSD (Hikari, EDAX, Inc., NJ, USA). Considering the grain size, the step size of EBSD was 3.5 μm.

After their initial states were observed, the specimens were subjected to secondary recrystallization at 1080°C at 5°C/min, held for 0 min, and transferred from the hot zone to the cold zone in the tube furnace. The interior of the tube furnace was placed under hydrogen (purity: 99.9999%) flow to prevent oxidization of the specimens. For time sequential observation of the abnormally-growing Goss grain, the specimens were heated sequentially for 11, 2.5, and 2.5 min, corresponding to a total of 16 min of heat treatment.

An oxide film is typically formed on Fe-3%Si grain-oriented electrical steel after heat treatment; this film must be removed to observe the ex-situ time evolution of the specimen [1,3]. The final polishing was carried out for 1 min using an alumina suspension (OP-S, Sturers, Inc., OH, USA), which removed less than 1 μm of the sample surface to eliminate the oxide film. Heat treatment at 1080°C and final stage polishing were repeated, and the ex-situ time evolution of the abnormally growing Goss grains was observed and analyzed by EBSD.

To investigate the CSL boundary relationship between the Goss grains and the matrix grains that remained or disappeared, each matrix grain was numbered, and the remaining or disappearing grains were examined according to the heat treatment time. The orientation of each grain was investigated to affirm the CSL boundary relationship.

## Ethics Statements

The authors declare that there are no ethical issues with the data presented and the methods used.

## CRediT Author Statement

**Tae-young Kim:** Conceptualization, Methodology, Validation, Investigation, Data curation, Writing – original draft, Writing - review & editing, Visualization, Supervision.

## Declaration of Competing Interest

The authors declare that they have no known competing financial interests or personal relationships that could have appeared to influence the work reported in this paper.

## Data Availability

Data on coincidence site lattice boundaries of abnormally growing Goss grains in Fe-3%Si steel (Original data) (Mendeley Data).
